# Current Status of Antimicrobial Drug Use in Japanese Companion Animal Clinics and the Factors Associated With Their Use

**DOI:** 10.3389/fvets.2021.705648

**Published:** 2021-09-24

**Authors:** Kohei Makita, Nami Sugahara, Kazuhiro Nakamura, Takeshi Matsuoka, Masato Sakai, Yutaka Tamura

**Affiliations:** ^1^Veterinary Epidemiology Unit, School of Veterinary Medicine, Rakuno Gakuen University, Ebetsu, Japan; ^2^Technical Support Department, MP Agro Co. Ltd., Tokyo, Japan; ^3^Japan Veterinary Medical Association, Tokyo, Japan; ^4^Center for Veterinary Drug Development, Rakuno Gakuen University, Ebetsu, Japan

**Keywords:** companion animal, antimicrobial drug use, antimicrobial resistance (AMR), Japan, sociological factors

## Abstract

The Japanese National Action Plan on Antimicrobial Resistance (AMR) was adopted to strengthen AMR surveillance and monitoring in companion animals. The Japanese Veterinary Antimicrobial Resistance Monitoring (JVARM) system monitors the sale of veterinary antimicrobial drugs by pharmaceutical companies, and the sale of human drugs by principal wholesale companies to companion animal (dogs and cats) clinics. However, the data do not include sales by local drug suppliers and personal importation to companion animal clinics in Japan. The purposes of this study were to estimate total antimicrobial drug use by companion animal clinics in Japan and to identify the factors associated with their use. In 2018, questionnaires gathering data on attributes of the clinic and volumes of antimicrobial drugs used were sent to 212 clinics across Japan by the Japan Veterinary Medical Association. Out of the clinics, 170 valid questionnaires were returned (80.2% response rate). Antimicrobial drugs were categorized first as human, veterinary, or imported drugs and then further categorized as important drugs (critically important drugs for humans and second-choice veterinary drugs) or others. Total antimicrobial drug use was estimated based on the number of clinics reported in 2016. The relationships between antimicrobial drug use and various questionnaire items were analyzed using non-parametric regression analysis. Total antimicrobial drug use was estimated at 29.9t, which was 2.1 times higher than reported by the JVARM survey on the sales of antimicrobial drugs. In terms of total use, important drugs and human drugs accounted for 12.6 and 61.8%, respectively. Clinic income per veterinarian was associated with total antimicrobial use per veterinarian. The proportion of important drugs among all antimicrobial drugs used in a clinic was high in recently established clinics with middle-aged and older directors.

## Introduction

Bacteria exhibiting antimicrobial resistance (AMR) represent a significant health threat to both humans and companion animals. The spread of AMR among bacteria in companion animal clinics reduces the efficacy of antimicrobial drugs for treating bacterial infections in animals. Companion animals may serve as carriers for bacteria exhibiting AMR (1). Infection in pet owners from their animals with antimicrobial resistant bacteria may result in the treatment failure. Infection of companion animals with antimicrobial resistant bacteria also poses risks for nosocomial infections to veterinarians, veterinary nurses, technicians, internship veterinary students, and other veterinary patients (animals) in the clinic. The World Health Organization proposed a Global Action Plan on Antimicrobial Resistance at the 68th World Health Assembly, and this plan was adopted in 2015 (2). The National Action Plan on AMR published in Japan in 2016 (3) suggested that surveillance and monitoring systems for AMR in companion animals would be strengthened. In 2017, antimicrobial resistant bacteria in companion animals were included in the Japanese Veterinary Antimicrobial Resistance Monitoring (JVARM) system (4).

The nature of the relationship between humans and their companion animals has changed over the last few decades. In Japan, the mean family size declined from 5.0 persons in 1953 to 2.5 in 2017 (5). For a comparison, the number of registered dogs was 1,905 thousand in 1960 (6), and the estimated number was increased to 8,903 thousand heads in 2018 (7). Based on data for 2016–2020, the estimated proportions of households rearing dogs and cats in Japan were 11.9% and 9.6%, respectively (8). Of the dogs and cats raised in 2016, 88.5% and 96.6% were raised inside the home (9), while those in 2004 were 60.7% and 73.5% (10), indicating that the relationship between humans and their companion animals has become closer. In developed countries, increasing attention to the welfare of companion animals has resulted in increased expenditures on veterinary care. Treatment of infectious disease includes antimicrobial therapy to bacterial infections, which can select antimicrobial-resistant bacteria (11). Antimicrobial-resistant bacteria of companion animal origin are now considered a hazard to human health (1). As early as 2003, it was pointed out that assessments of the risk of AMR to human health are limited by a heavy focus on food animals, neglecting AMR originating in companion animals (12). A 2002 report argued that there were warning signs of AMR in canine medicine and that prudent use of antimicrobial drugs and the development of science-based infection control practices were needed (13). A large number of more recent studies has described the common colonization of pet-owning household members and their animals with antimicrobial-resistant bacteria (14, 15).

The most important factors affecting the emergence of antimicrobial -resistant bacteria are misuse and overuse of antimicrobial agents (16). Therefore, in terms of planning for effective AMR control, it is critically important to monitor the amount of antimicrobial drugs used. However, there are limited data regarding antimicrobial drug use in companion animals globally. In Japan, the only available regular data of antimicrobial drug use in animals were annual sales of antimicrobial drugs for animals by principal pharmaceutical companies that have been approved by the government, and reported by the JVARM (17). A 2016 study examined the amount of antimicrobial drugs designated for use in humans that were sold to companion animal clinics (18). That study revealed that a large amount of antimicrobial drugs approved for humans are used in companion animal medicine. The JVARM data do not include personally imported antimicrobial drugs and the sale of antimicrobial drugs for use in companion animals by retail drug suppliers. Therefore, accurate amount of antimicrobial drug use for companion animals in Japan is not known.

To overcome the challenges associated with monitoring antimicrobial drug use in companion animal clinics, the Japan Veterinary Medical Association (JVMA) conducted a survey using structured questionnaires focused on antimicrobial drug use in companion animal veterinary clinics. The objective of the present study was to elucidate the current trends and factors associated with antimicrobial drug use in these veterinary clinics based on survey data for promoting the prudent use of antimicrobials.

## Materials and Methods

### Questionnaire Survey

The target population included companion animal (dogs and cats) clinics belonging to the local Veterinary Associations in all the prefectures of Japan. There were 11,839 companion animal clinics in Japan in 2016 (19). The total sample size of 200 clinics was proportionally allocated among all prefectures according to the number of clinics reported. The JVMA has been conducting rectal swab sampling from healthy pet animals for AMR monitoring at 200 veterinary clinics, requested by the Ministry of Agriculture, Forestry and Fisheries (MAFF), under the JVARM program (4). The JVMA decided the sample size of 200, conforming to the monitoring scheme. The clinics participating in the monitoring scheme and this study are not matched.

Questionnaires were sent to 212 companion animal clinics on July 25, 2018, with a requested return by August 15, 2018. The responses in the questionnaires were digitized by the JVMA, and anonymized data were provided to the researchers for the analysis. Survey questions addressed attributes of the clinic and the director (respondents), such as prefecture and city/town/village, age, years in operation since clinic establishment, corporation or not, primary/secondary clinics, annual clinic income, and number of veterinarians and other staff. In the current study, the number of veterinarians but that of other staff was used for the analysis. A companion animal clinic is registered as either sole proprietorship or corporation in Japan. Questions also covered antimicrobial drug use, such as category of veterinary, human, and imported drugs used, commercial name, ingredient amount, and annual volume used between April 1, 2017 and March 31, 2018. Veterinary and human drugs refer to those approved by MAFF and the Ministry of Health, Labour, and Welfare (MHLW) of Japan, respectively. Imported drugs are those personally imported drugs. Regardless the designation to either veterinary or human drugs in the exporting country, personally imported drugs were categorized as imported drugs.

### Statistical Analysis

To ensure the representativeness of the clinics that responded with regard to the Japanese situation, Spearman's correlation test was used to assess the correlation between the number of companion animal clinics by prefecture reported to the government in 2016 (19) and those that responded to this study.

For descriptive analyses, the mean, median, and interquartile range were calculated for the age of directors, years in operation since establishment, annual clinic income, and number of veterinarians. The proportions of primary- and secondary-level (referral) clinics in corporation clinics, and those in non-corporation (sole proprietor) clinics were calculated. The proportion of corporation clinics was compared with 50% (equal probability) using 1 sample chi-square test.

Descriptive statistics were also performed regarding antimicrobial drugs used. In this study, important antimicrobial drugs in companion animal medicine were defined based on whether they are listed as rank I antimicrobial drugs for human which target bacteria that affect human health through food items, according to the Food Safety Commission (20), or are second-choice veterinary drugs approved by the MAFF of Japan, as of March 2018 (21). The second-choice veterinary drugs refer to those must be used after confirming by microbiological tests that a first-choice veterinary drug is not effective. The rank I antimicrobial drugs for human included 14- and 15-membered-ring macrolides, kanamycin, carbapenems, glycopeptides, 3rd generation cephalosporins, oxacephems, polypeptides (colistin), fluoroquinolones, and mupirocin. The list of rank I antimicrobial drugs include all the highest priority drugs in the WHO Critically Important Antimicrobials for Human Medicine: cephalosporines, glycopeptides, macrolides, polymixins (colistin), and quinolones (22). The rank I antimicrobial drugs were also chosen, as they are well-recognized in Japan. The second-choice veterinary drugs included fluoroquinolones (enrofloxacin and orbifloxacin), 3rd generation cephalosporins (cefmenoxime, cefpodoxime, and cefovecin), and 15-membered-ring macrolides. First, the total amounts of veterinary, human, and imported drugs were calculated. Second, the total amounts by drug type were calculated. Third, the proportion of important antimicrobial drugs relative to total antimicrobial drug use was calculated for each clinic. Total antimicrobial drug use in Japan was estimated by dividing the total amount used in the clinics studied by the number of responding clinics, multiplied by the total number of clinics in Japan (*n* = 11,839) (19).

The factors associated with antimicrobial drug use were analyzed using data from 148 clinics (70.0%, 148/212) that provided responses to all survey questions. Three objective variables were selected: (i) total annual antimicrobial drug use per veterinarian, (ii) the proportion of rank I antimicrobial drugs relative to the total annual antimicrobial use in the clinic, and (iii) the proportion of important antimicrobial drugs that included rank I antimicrobial drugs and second-choice veterinary drugs. Table 1 shows the explanatory variables examined to assess the relationship with antimicrobial drug use patterns. Information regarding population and area were collected from 2017 MHLW vital statistics (23), and the Ministry of Land, Infrastructure, Transport and Tourism prefectural and municipal aerial survey (24). The numbers of dogs registered and vaccinated against rabies for each prefecture were collected from MHLW data (25).

**Table 1:** Variables examined for the statistical analysis of the relationship with antimicrobial drug use.

**Category**	**Variable**
Socio-economic attributes of the clinic	(1) Region: Hokkaido and Tohoku, Kanto, Chubu, Kinki, Chugoku-Shikoku, Kyushu-Okinawa; (2) population density; (3) registered dog density; (4) density of veterinary clinics by prefecture; (5) rabies vaccination rate by prefecture; and (6) located or not in ordinance-designated city
Attributes of the director	(7) Director age in years and (8) years clinic has been in operation by the director
Management style	(9) Number of veterinarians; (10) clinic income per veterinarian; (11) corporation or not; and (12) operation of secondary clinics

For the selection of statistical models, the error structures for the outcome variables were examined. Total annual antimicrobial use per veterinarian was not normally distributed, as determined by the Shapiro test. It also could not be transformed into a normal distribution via Box-Cox transformation because of the strong skew. The proportion of rank I antimicrobial drugs and important antimicrobial drugs at many clinics included zero and thus could not be transformed into logit form (logarithm cannot be taken for zero). Therefore, non-parametric regression analyses were employed using the np package (26). After univariable analyses for all the explanatory variables (Table 1), a bivariable analysis was performed selecting explanatory variables as those with *p* < 0.05 and a potential confounder, for the proportion of important drugs. A confounder was identified by qualitative discussion of causality after the univariable analyses. The relationships between explanatory variables were examined using linear regression. All the statistics were performed using statistical software R, version 3.6.1 (27).

## Results

### Attributes of the Responding Clinics

The response rate for the 212 clinics across Japan was 86.8% (184 clinics). Excluding questionnaires that lacked information on the amount of active ingredient used, the valid response rate was 80.2% (170/212). Of the 170 clinics that provided valid responses, one clinic responded only the questions about antimicrobial drug use.

**Figure 1:**
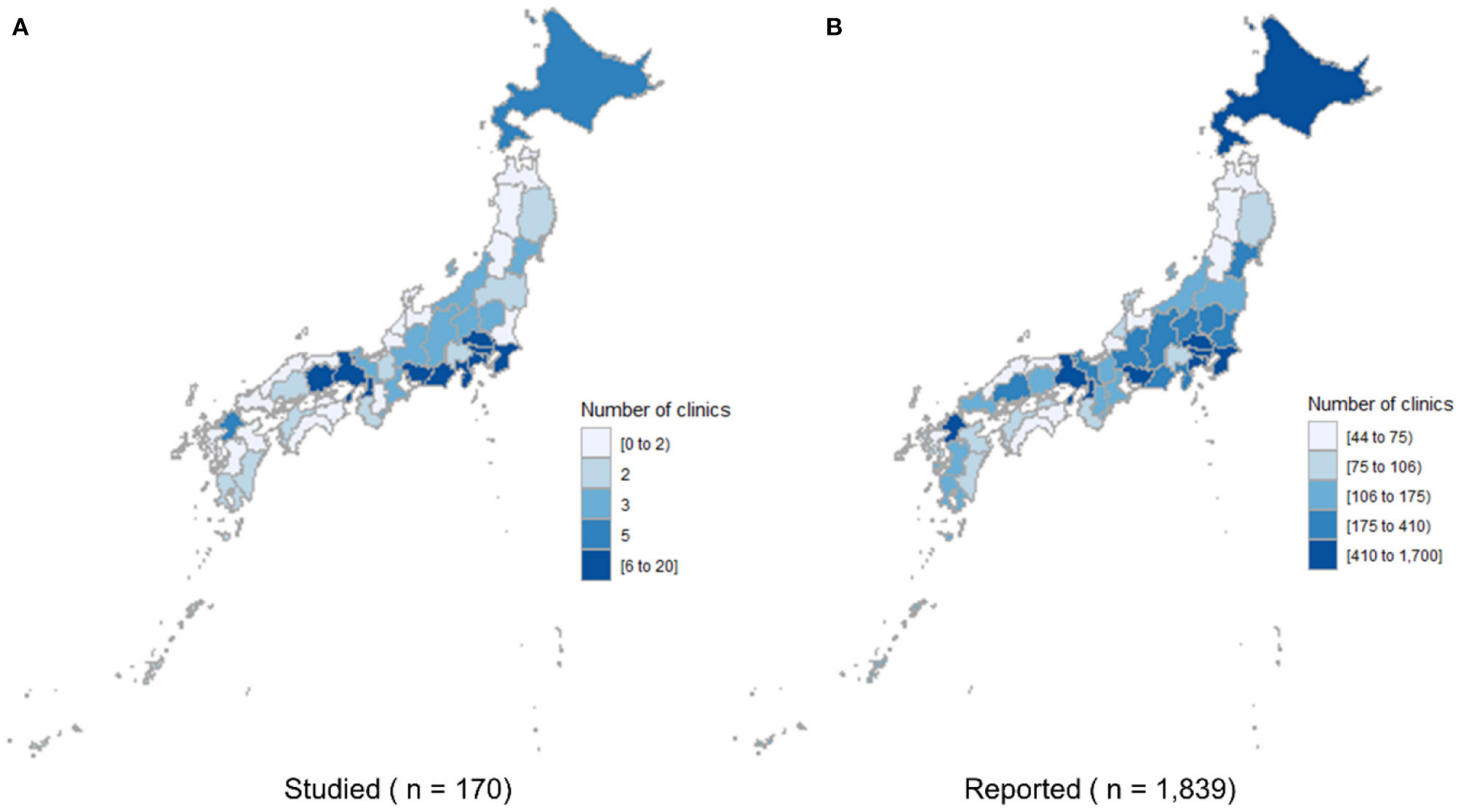
Choropleth maps showing the number of companion animal clinics studied **(A)** and the number reported to the government **(B)** by prefecture.

The number of companion animal clinics that responded and the number reported to the government by the prefecture were significantly correlated (correlation coefficient = 0.85, *p* < 0.01, Figure 1). The 170 clinics that provided valid responses accounted for 1.4% of the total number of clinics in Japan (*n* = 11,839).

The mean, median, interquartile range, and range of clinic director age were 52.4, 53, 46.8–58.0, and 34–74 years, respectively. The mean, median, interquartile range, and range of years in operation were 21.2, 22, 13.0–29.0, and 1–47 years, respectively. The mean, median, interquartile range, and range of annual total clinic income were 93.1, 60.0, 37.0–105.0, and 5.0–1000.0 million Japanese Yen (JPY), respectively. They are equivalent to 844.6, 544.3, 335.7–952.6, and 45.4–907.2 thousand US dollars (1 US dollar = 110.23 JPY as of August 7, 2021). The mean, median, interquartile range, and range of number of veterinarians in the clinic were 4.2, 3, 2–4, and 1–57, respectively.

Table 2 shows the type of management and operation of 169 clinics that provided valid responses. The largest number of clinics that responded was primary corporation clinics (57.4%, 97/169). There were significantly more corporation-style clinics (66.3%, 112/169) than non-corporation clinics (33.7%, 57/169, *x*^2^ = 17.3, df = 1, *p* < 0.001). The majority of secondary clinics were corporations (78.9%, 15/19).

**Table 2:** Attributes of responding clinics by management style and type of operation.

	**Type of operation**		
**Management style**	**Primary**	**Secondary**	**Total**
Corporation	97 (57.4%)	15 (8.9%)	112 (66.3%)
Sole proprietorship	53 (31.4%)	4 (2.4%)	57 (33.7%)
Total	150 (88.8%)	19 (11.2%)	169

### Antimicrobial Drug Use at the Companion Animal Clinics Studied

Table 3 shows the amounts of various antimicrobial drugs used by drug category at the companion animal clinics studied, the amounts estimated for all Japanese clinics, and the proportions of drugs relative to all usage. In total, an estimated 29.9t of antimicrobial drugs were used at Japanese companion animal clinics between April 1, 2017 and March 31, 2018. Drugs were categorized as unknown, when the brand name and/or status of importation were not provided. The largest proportions of antimicrobial drugs used were antimicrobial drugs for human medicine (61.8%) and veterinary drugs (36.7%). Imported antimicrobial drugs accounted for 1.5%.

**Table 3:** Amounts of various antimicrobial drugs used annually at the companion animal clinics studied and estimated amounts used at Japanese clinics, by drug category.

**Category**	**Total amount of drugs used at clinics studied (kg)**	**Estimated amount of drugs used at Japanese clinics (kg)**	**Proportion (%)**
Veterinary drugs	157,763	10,986,801	36.7
Human drugs	265,665	18,501,223	61.8
Imported drugs	6,263	436,163	1.5
Unknown	30	2,089	<0.1
Total	429,721	29,926,276	100.0

The mean, median, interquartile range, and range of annual amount of antimicrobial drugs used per veterinarian were 694.7, 466.0, 271.0–800.0, and 38.0–5,023.9 kg, respectively.

Table 4 shows the total annual amounts used at the studied clinics according to antimicrobial drug type. The main antimicrobial drugs used belonged to beta-lactam class (penicillin, 1st, 2nd, and 3rd cephalosporins, oxacephem, carbapenem, and penem), which accounted for 78.1% of total usage. The 1st generation cephalosporin (41.1%) and penicillin (33.6%) were top two commonly used antimicrobial drugs. Cefalexin accounted for 95.5% of 1st generation cephalosporin, and the rest was cefazolin. Amoxicillin was the most used penicillin drug (83.6%), followed by ampicillin (12.8%). Benzyl penicillin potassium accounted only for 1.8% among penicillins used, and the majority of penicillins was with extended spectrum. When the annual amount of drug used was calculated based on drug type, beta-lactam antimicrobial drugs (penicillin, cephalosporins, oxacephem, carbapenem, and penem) were the most used (78.1%), followed by fluoroquinolones (8.0%). The other drugs listed in Table 4 include fosfomycin (8,488.0 g), fucidin (49.4 g), and mupirocin (0.3 g).

**Table 4:** Total annual amount used (in grams) at the studied clinics categorized by antimicrobial drug type, and the percentage of the total drugs used.

**Antimicrobial drug type**	**Total annual amount (g)**	**Percentage (%)**
Penicillin	144,318	33.6
First-generation cephalosporin	176,531	41.1
Second-generation cephalosporin	1,378	0.3
Third-generation cephalosporin	10,849	2.5
Oxacephem	40	0.0
Carbapenem	382	0.1
Penem	1,943	0.5
Aminoglycoside	1,362	0.3
Macrolide	11,963	2.8
Lincomycin	7,083	1.6
Tetracycline	13,375	3.1
Amphenicol	5,927	1.4
Sulfamethoxazole/trimethoprim	11,814	2.7
Fluoroquinolones	34,244	8.0
Polypeptides	13	<0.1
Glycopeptides	22	<0.1
Others	8,538	2.0

**Figure 2:**
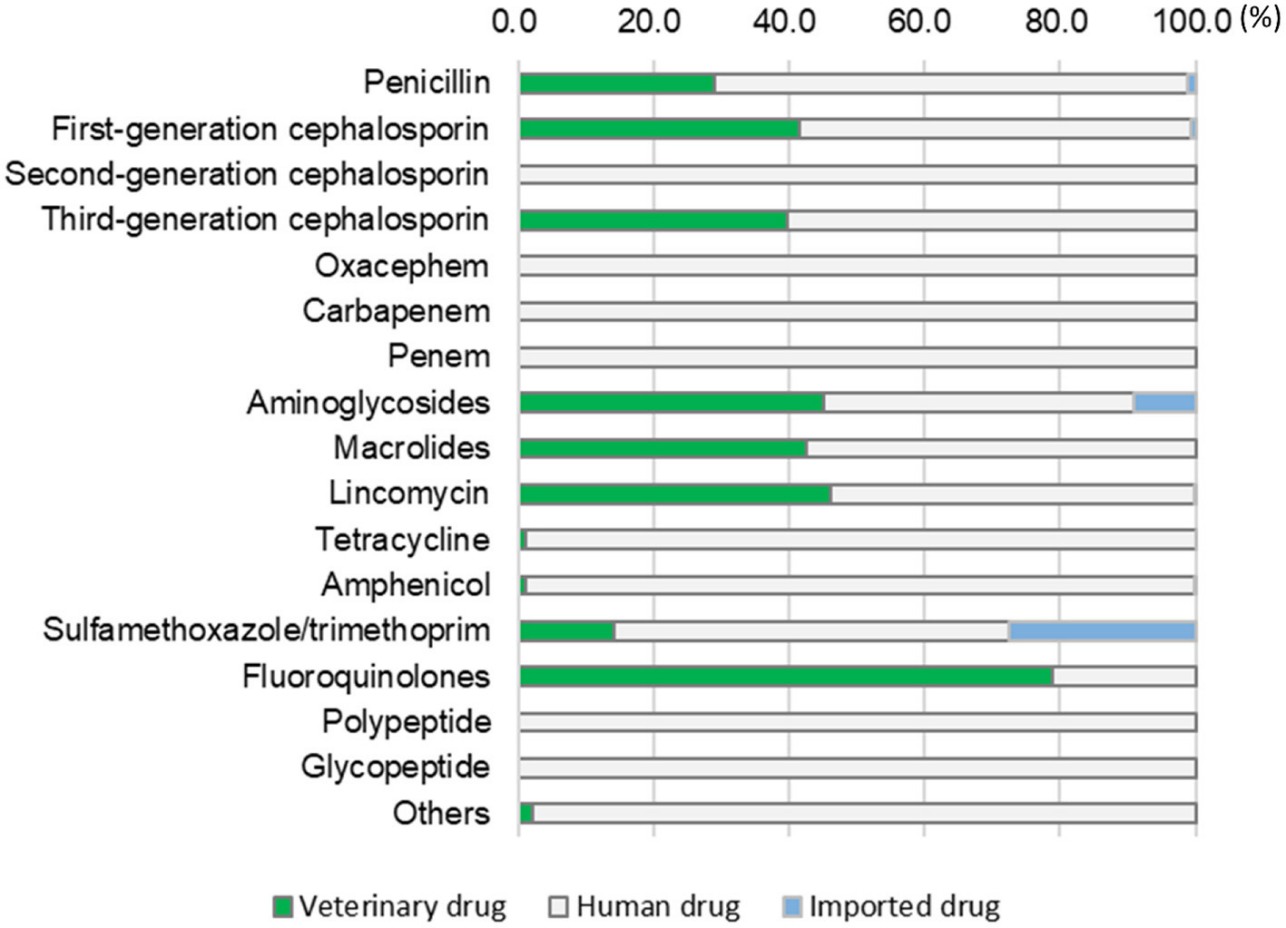
Proportions of antimicrobial drugs used in the studied clinics, categorized by drug type (veterinary, human, or imported).

Figure 2 shows the proportions of veterinary, human, and imported drugs among the antimicrobial drugs listed in Table 4. Human drugs accounted for more than half of all use in each category, except for fluoroquinolones (21.1%). The human drugs used were exclusively those produced for human medicine, such as 2nd generation cephalosporin, oxacephem, carbapenem, penem, polypeptides (colistin), and glycopeptides (vancomycin) (veterinarians can use such drugs unless they are prohibited bylaws). However, only low amounts of these drugs were used (Table 4). Imported drugs accounted for a high proportion of all use in the sulfamethoxazole/trimethoprim category (27.6%), followed by aminoglycosides (9.0%, Figure 2).

The proportions of rank I antimicrobial drugs and second-choice animal drugs among the total amount of antimicrobial drugs used at the 170 clinics studied were 4.3 and 8.3%, respectively. The total proportion of important drugs defined in this study was 12.6% (rank I and second-choice veterinary drugs were mutually exclusive).

**Figure 3:**
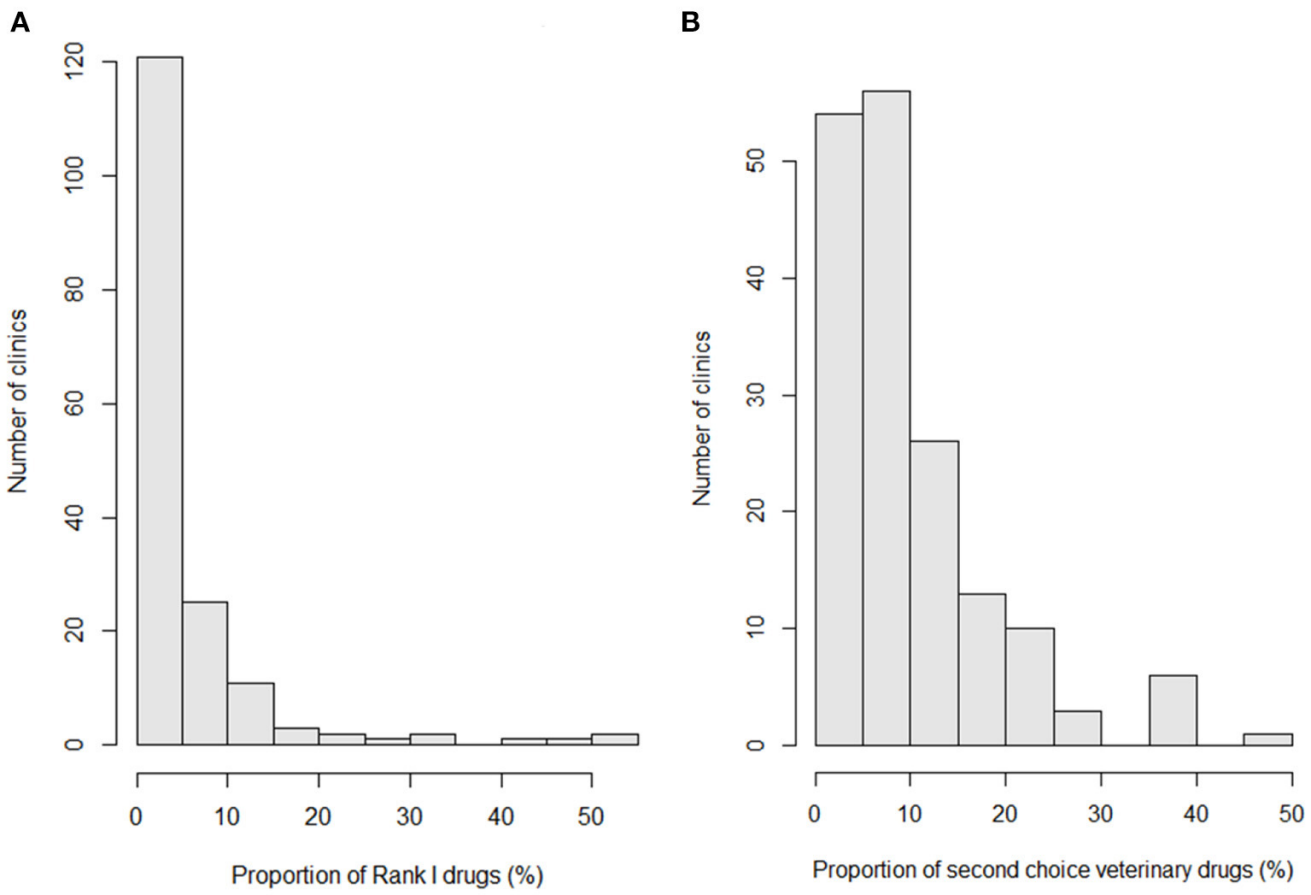
Histograms indicating the proportions of rank I **(A)** and second-choice veterinary drugs **(B)** used at the studied clinics.

Figure 3 shows the proportions of rank I (a) and second-choice veterinary drugs (b) used at the studied clinics. The mean, median, interquartile range, and range of the proportion of rank I drugs among the total amount of antimicrobial drugs used at a clinic were 4.8, 1.4, 0.1–5.9, and 0–53.6%, respectively (Figure 3A). The rank I drugs were heavily used at a small number of clinics. The mean, median, interquartile range, and range of the proportion of second-choice veterinary drugs among the total amount of antimicrobial drugs used at a clinic were 9.9, 7.4, 4.4–12.3, and 0.4–45.2%, respectively (Figure 3B). These drugs were more commonly used than rank I drugs. Similarly to the rank I drugs, small number of clinics heavily used second-choice veterinary drugs. The correlation between the proportion of rank I drugs and proportion of second-choice veterinary drugs was poor (correlation coefficient = 0.002, *p* = 0.98). The mean, median, interquartile range, and range of the proportions of important drugs (rank I and second-choice veterinary drugs) among the total amount of antimicrobial drugs used at a clinic were 14.8, 11.3, 7.1–18.7, and 0.4–66.4%, respectively.

### Factors Associated With Antimicrobial Use

**Figure 4:**
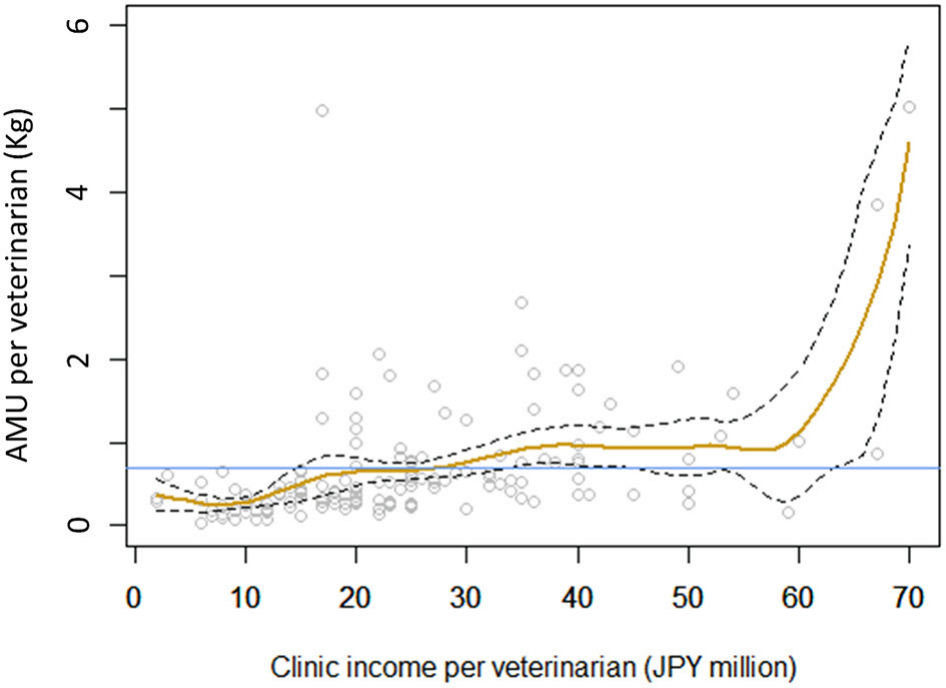
Relationship between clinic income per veterinarian and antimicrobial drug use per veterinarian (in milligrams). Yellow line shows the fitted line, and broken lines show the 95% confidence interval. Blue line shows the mean amount of antimicrobial drugs used among the clinics studied.

Among the various socio-economic factors studied, only one variable, clinic income per veterinarian, was significantly associated with the amount of antimicrobial drugs used per veterinarian (*p* = 0.018). The antimicrobial use per veterinarian was lower than the mean (764 g) at clinics with an income per veterinarian of less than JPY 28 million (US dollars 254 thousand). Antimicrobial use was very high at clinics with an income per veterinarian of greater than JPY 60 million (Figure 4).

**Figure 5:**
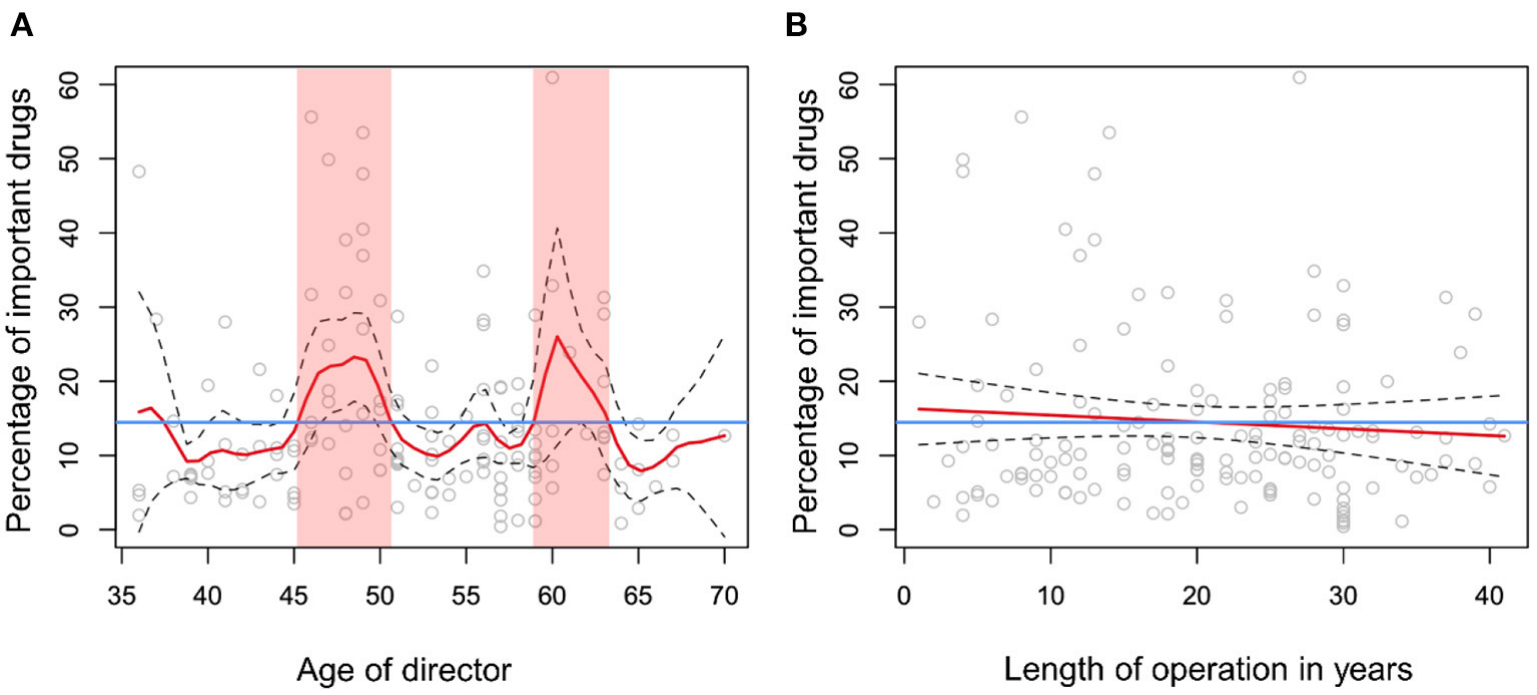
Relationships between the proportion of important drugs among all antimicrobial drugs used and **(A)** age of the clinic director (years), and **(B)** years clinic has been in operation. Red lines in panels **(A,B)** show the fitted non-parametric regression lines. Blue lines in panels **(A,B)** show the mean percentage of important drugs relative to total usage, 14.2%. Red ribbons in panel **(A)** indicate the age ranges where percentage of important drugs exceeded the mean value.

No factors were significantly associated with the proportion of rank I antimicrobial drugs among all antimicrobial drugs used at a clinic, according to non-parametric regression analysis. When rank I antimicrobial drugs and second-choice veterinary drugs were combined as important drugs, only one significant factor was identified, age of the clinic director (*p* = 0.003, Figure 5A). The following age groups were associated with use of important drugs at a higher proportion than the mean (14.0%) among all antimicrobial drugs used at a clinic: <37, 45–51, and 59–63 years (Figure 5A). As the age of the director was significantly associated with the time clinic has been in operation (in years) according to linear regression analysis (slope = 1.13, standard error = 0.04, *p* < 0.001), these two variables were included in the bivariable non-parametric regression analysis. In this analysis, years in operation was not associated with the proportion of important drugs among antimicrobial drugs used at a clinic (*p* = 0.37, Figure 5B).

**Figure 6:**
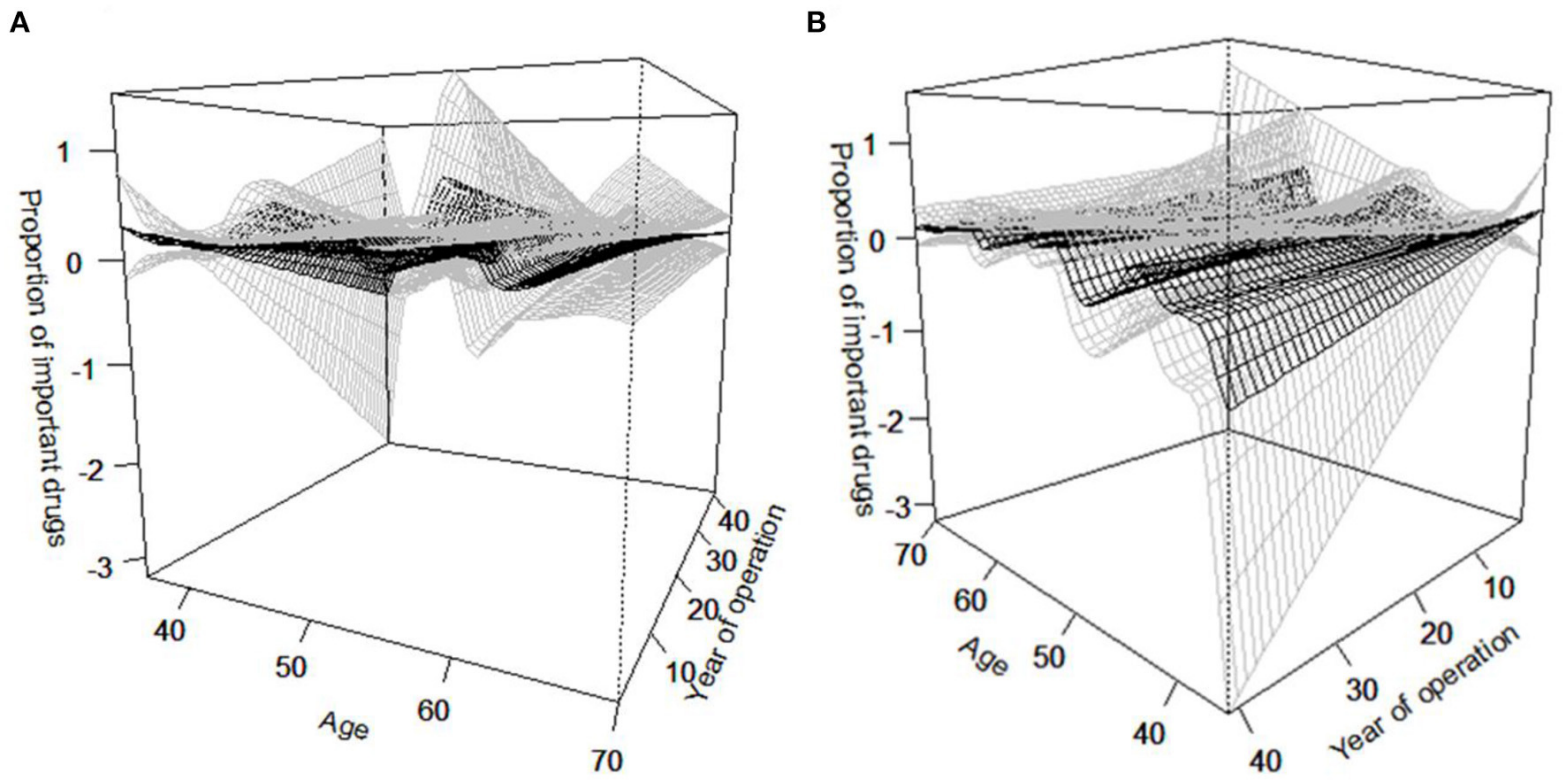
3D depiction of relationships between the proportion of important drugs among all antimicrobial drugs used, age of the clinic director, and years in operation shown from different angles. The relationships are shown from different angles to understand the factors associated with the proportion of important drugs in terms of **(A)** age of director, and **(B)** years of operation associated with the age of director.

In the bivariable non-parametric regression analysis, 3D-plots showed that in the age groups 45–51 and 59–63 years, clinics with shorter years in operation used higher proportions of important drugs among all antimicrobial drugs used at those clinics (Figures 6A,B). The upper limit of the confidence interval for the proportion of important drugs used at clinics where the director was 60 years old was particularly high when the years in operation was short (Figure 6A), reflecting an outlier (Figure 5A).

## Discussion

To the best of our knowledge, this is the first study to describe the status of antimicrobial drug use at companion animal clinics in Japan. The estimated amount of antimicrobial drugs used at companion animal clinics in Japan (29.9t) was 2.1 times greater than that reported by the 2016 MAFF survey (14.3t) based on the sales of human and veterinary antimicrobial drugs (18). Moreover, the amount of human antimicrobial drugs estimated as being used at companion animal clinics in this study (18.5t) was 2.8 times greater than the survey results regarding sales volume (6.5t) (18). There are several reasons why the estimated amount of antimicrobial drugs used was far greater than the sales volume. First, the sales volume study targeted only direct sales from primary wholesale companies to animal clinics. This figure does not include the sales to animal clinics by local drug suppliers. Second, the sales survey cannot account for actual use in cases in which clinics purchase antimicrobial drugs in bulk before the year of survey. In addition, this study showed that the proportion of imported drugs appears to be high. The amount of imported drugs used in companion animal clinics was not included in previous studies. According to our personal communications with veterinary clinicians, purchasing imported drugs via the internet is common.

Since publication of the National Action Plan on AMR in Japan in 2016 (3), recognition of the importance of prudent use of antimicrobial drugs has increased. The data on the sales volume of human drugs in companion animal clinics have not been published, aside from that published in 2016 (18), and accurate trend is not known for that. However, the sales volume of veterinary drugs to companion animal clinics by pharmaceutical companies have been published since 2005 (28). These data indicate a decreasing trend in sales; sales from 2013 to 2017 were 9.6, 8.1, 8.9, 7.8, and 6.8t. Therefore, the volume of sales of human drugs to companion animal clinics in 2018, when this study was conducted, might have been lower than in 2016 (18), and the ratio of drugs used to sales reported might be lower than 2.8 times greater.

This study used total reported use of antimicrobial drugs in milligrams as the unit of antimicrobial use. These units have been used to describe the level of antimicrobial use in companion animals (in kilograms) (29). However, use of these units has some limitations. For example, effective doses in milligrams differ between drugs, and the level of prudent use of an antimicrobial drug cannot be compared between sectors or countries, as the number of animals of a given species in a country and the size of animals by species and breed differ. For food animals, species-specific coefficients of antimicrobial consumption per population correction unit are used to compare levels of antimicrobial drug use between livestock species (30). The European Medicines Agency, European Surveillance of Veterinary Antimicrobial Consumption group introduced the veterinary defined daily dose (DDD_VET_) criterion to objectify the numerator in 2016 (31). DDD_VET_ is similar to the defined daily dose (DDD) in human medicine, which is the assumed average maintenance dose per day for a drug used for its main indication in adults (32). Future studies on antimicrobial drug use in companion animals should consider using DDD_VET_ to facilitate between-clinic and between-country comparisons.

In the present study, clinic income per veterinarian was positively associated with antimicrobial drug use per veterinarian. Another characteristic of antimicrobial drug use was that the proportion of important drugs among antimicrobial drugs used was bimodal, high among the middle-age and older groups with fewer years since clinic establishment. Regarding the amount of antimicrobial drugs used per veterinarian, there were no detailed questions on clinic specialty in the questionnaire, and we could not find any notable reason to explain this relationship. Higher frequency of prescription of antimicrobial drug may be the reason of higher income per veterinarian. Referral hospitals have more complex care services than smaller clinics. However, the amount of antimicrobial use per veterinarian was not different between two groups. In our study, detailed drug prescription and price data were not collected, and this hypothesis remains unanswered. A few clinics heavily used rank I antimicrobial drugs, but no factor explaining this observation was identified. There was no correlation between the proportions of rank I antimicrobial drugs and second-choice drugs relative to the total amount of antimicrobial drugs used. The heavy users of rank I antimicrobial drugs did not necessary used second-choice veterinary drugs heavily. The types of second-choice veterinary drugs (fluoroquinolones, 3rd generation cephalosporins, and 15-membered-ring macrolides) are included in the rank I antimicrobial drugs (for humans). The clinics may be choosing either type they prefer, and fix to the selection. The reasons for the bimodal distribution among middle-aged and older directors with fewer years since clinic establishment in using a high proportion of important drugs could be related to financial needs or a need to ensure reliable efficacy of the drugs. However, the reasons remain unknown, as questions regarding the motivation behind antimicrobial use were not asked. Previous studies suggested several factors as potentially influencing antimicrobial drug use in companion animal clinics. A qualitative study in the UK suggested that factors other than clinical evidence and scientific knowledge, such as perceived efficacy, ease of administration, and pet owners' willingness and ability to treat, influence antimicrobial usage (33). More recently, antimicrobial stewardship efforts began to demonstrate effects among both practitioners and clients. Client decisions focused on preventive health, vaccination, insurance, neutering of dogs, and practices accredited by the Royal College of Veterinary Surgeons were associated with decreased odds of systematic antimicrobial prescriptions for dogs and cats (34).

In Japan, the use of antimicrobial drugs in food animals appears to be prudent. The proportions of 3rd generation cephalosporins and fluoroquinolones, which are important in human medicine, among total sales of antimicrobial drugs used for food animals between 2013 and 2016 were <1% (35). In contrast, the proportion of second-choice veterinary drugs used in companion animal clinics in this study was 8.3%. The most common veterinary drug used in food animals in Japan in 2017 was tetracyclines (40% of total sales), followed by macrolides (16%) (36). The most common antimicrobial drugs used for companion animals in this study were 1st generation cephalosporin (41.1%) and penicillin (33.6%, of which 98.2% was extended spectrum). In Japanese human medicine, the most commonly used antimicrobial drug in 2019 was macrolides (14.5%), followed by 3rd generation cephalosporins (14.2%), and penicillin with extended spectrum (10.4%) by sales volume (37). In Denmark, the most commonly used antimicrobial drugs for pet animals in 2019 were penicillin with extended spectrum (27.3%), followed by sulfonamides and trimethoprim (20.8%) (38). The proportion of cephalosporins relative to all antimicrobial drugs used for pets was 7.9%. The majority (98.8%) of cephalosporines used for pets was 1st or 2nd generation cepharosporins in the country (38). The estimated numbers of dogs and cats raised in 2018 were 8,903 and 9,649 thousand heads in Japan (7), and 595 and 675 thousand heads in Denmark (39), respectively. Using them, the average annual volumes of antimicrobial drugs used per dog or cat in 2018 were 1.61 g in Japan (total 29.9t in this study), and 0.96 g [total 1,224 kg in DANMAP report (38)] in Denmark. From these figures, Japanese companion animal clinics can reduce total amount of antibiotics greatly, particularly important drugs for humans such as cephalosporines.

In the AMR monitoring survey of sick pet animals in Japan in 2017, the rates of *Escherichia coli* resistance to cefotaxime (a 3rd generation cephalosporins) and ciprofloxacin (a fluoroquinolone) were 26.1 and 43.2% in dogs and 33.8 and 39.0% in cats, respectively (4). AMR monitoring of healthy pet animals started in 2018. In 2018, the rates of *E. coli* resistance to aminobenzyl penicillin, cefotaxime and ciprofloxacin were 33.8, 13.2, and 18.5% in dogs, and 28.5, 10.8, and 12.0% in cats, respectively (37). In contrast, rates of resistant *E. coli* from healthy cattle, pigs, and broilers to cefotaxime in 2017 were 0.4, 1.2, and 4.7%, respectively, and rates of resistance to ciprofloxacin were 0, 0, and 12%, respectively (36). Low rates of *E. coli* resistance were observed in sick food animals in 2017 as well: 1.7 and 0% resistance to cefotaxime in cattle and pigs and 1.7 and 4.5% resistance to ciprofloxacin (36). The practice of prudent use of antimicrobial drugs in companion animal medicine in Japan requires much improvement. Currently, use of antimicrobial drugs for humans in companion animals is not prohibited in Japan. Additional guideline may be needed to facilitate the change in drug use.

To control the use of antimicrobial drugs, monitoring may be the first step. The rapid decrease in Danish national pig antimicrobial consumption was observed after the introduction of “Yellow Card Scheme” in 2010 (40). This was achieved because all antimicrobial drugs prescribed for use in animals have been collected in the national database VetStat since 2000, and monitored by the Danish Integrated Antimicrobial Resistance Monitoring and Research Programme (DANMAP) (41). AMR monitoring was not the only factor for reducing the antimicrobial drug use in Denmark, but the penalty against overuse (42). The monitoring system in pets and horses are not as complete as for production animals in DANMAP. Antimicrobial agents registered by either the pharmacy or veterinarians for use in companion animals are entered into VetStat without defining animal species (41). However, the data allow estimating the amount of drug use in pets and horses in Denmark (38, 41). Similarly, all pharmacies in Sweden are obliged to report all sales of medical and veterinary medicinal products to the eHealth Agency. In this system, sales from pharmacies to animal owners who were dispensed prescriptions, or to veterinarians upon requisition are recorded (43). In the EU, by the EU regulation 2019/6, obligatory monitoring of antimicrobial drug use in dogs and cats will start in 2029 (44). In Japan, antimicrobial drugs for dogs and cats are almost exclusively dispensed in animal clinics. Therefore, in this study, the antimicrobial drug use in dogs and cats in Japan was characterized. However, such information cannot be obtained annually at the moment, as monitoring scheme of antimicrobial drug prescription for pets is not in place. Therefore, introduction of monitoring system based on prescription should be considered. Moreover, epidemiological researches on the commonly diagnosed bacterial diseases, antimicrobial susceptibility, and treatment patterns in companion animal clinics will elucidate the magnitude of unnecessary use of antimicrobials.

Concomitant with monitoring, communicating the importance of prudent use of antimicrobial drugs in companion animal clinics to veterinarians and pet owners via university education programs and seminars is also necessary. The present study demonstrated that the prudent use of antimicrobial drugs may be practiced in the majority of clinics in Japan. On contrary, a small proportion of clinics are heavy users of critically important antimicrobial drugs for humans. However, this study did not attempt to evaluate the appropriateness of antimicrobial drug use. Future studies should examine the therapeutic decisions at bacterial infection cases. We hope that this information is taken seriously across Japan and used to increase awareness regarding the risks of selecting antimicrobial-resistant bacteria in companion animals, which would reduce the use of antimicrobial drugs in companion animal clinics in Japan.

## Conclusion

The study characterized the antimicrobial drug use in companion animal clinics in Japan for the first time. The prudent use of antimicrobial drugs may be practiced in the majority of companion animal clinics, but a small proportion of clinics are heavy users of critically important antimicrobial drugs. The study provides important information in raise awareness about prudent use of antimicrobial drugs in Japanese companion animal clinics. Currently, monitoring of detailed information of antimicrobial drug use in companion animal clinics is not in place, and introduction of monitoring scheme based on prescription should be considered.

## Data Availability Statement

The raw data supporting the conclusions of this article will be made available by the authors, without undue reservation.

## Ethics Statement

This study was approved by the Japan Veterinary Medical Association. The information was collected using a postal survey and informed consent was obtained in the form of opt-out. The data had been anonymized for the analysis.

## Author Contributions

KN, TM, MS, and YT conceived the study and developed the methodologies and questionnaire. TM collected the field data. KM and NS conducted statistical analysis. KM, NS, and YT wrote the manuscript. All authors listed have made a substantial, direct and intellectual contribution to the work, and approved it for publication.

## Funding

This study was funded by the Japan Veterinary Medical Association.

## Author Disclaimer

The findings and conclusions contained in this manuscript are those of the authors and do not necessarily reflect positions or policies of the Japan Veterinary Medical Association.

## Conflict of Interest

KN is employed by MP Agro Co. Ltd. The remaining authors declare that the research was conducted in the absence of any commercial or financial relationships that could be construed as a potential conflict of interest.

## Publisher's Note

All claims expressed in this article are solely those of the authors and do not necessarily represent those of their affiliated organizations, or those of the publisher, the editors and the reviewers. Any product that may be evaluated in this article, or claim that may be made by its manufacturer, is not guaranteed or endorsed by the publisher.
