# Supplementary material for: Current Status of Antimicrobial Drug Use in Japanese Companion Animal Clinics and the Factors Associated With Their Use
Source: Front Vet Sci. 2021 Sep 24;8:705648. doi: 10.3389/fvets.2021.705648 (PMC8500396; doi:10.3389/fvets.2021.705648)
Supplement: Supplementary file 1 [file Data_Sheet_1.PDF]

(Question/response sheet)

A survey on situations of antimicrobial drugs (veterinary, human, and imported drugs) and microchip

2018 Japan Veterinary Association

A. Questions for the clinic and staff

(Please check a circle to appropriate selection, or provide numbers in spaces with underline for each question)

【Director of the clinic】

Question 1. Sex 1. Male, 2. Female

Question 2. Age________years old

Question 3. The year since establishment of the companion animal clinic ____years

Question 4. Management style of the clinic 1. Corporation, 2. Sole proprietor

Question 5. Operation style of the clinic (multiple answers allowed)

1. Primary care 2. Visiting patient 3. Secondary (specialized) care 4. Night call

Question 6. Total income of domestic animal clinic of one year between 2017 April 1 and 2018 March 31 Approximately_______Yen

Question 7. Animal species seeing (multiple answers allowed)

1. Dog 2. Cat 3. Pet bird 4. Rabbit 5. Hamster/guinea pig/ferret 6. Reptile/amphibian 7 Fish

【Staff】

Question 8. Number of staff

Veterinarian Male____ Female ___

Qualified veterinary nurse Male____ Female____

Other veterinary support staff than qualified nurse Male_____ Female_____

Other staff (trimer, receptionist) Male____ Female____

Question 9. Age of staff according to the occupation

Veterinarian Mean ____ range (____ to ____)

Qualified veterinary nurse Mean ____ range (____ to ____)

Other veterinary support staff than qualified nurse Mean ___ range (____ to ___)

Other staff (trimer, receptionist) Mean ____ range (____ to ____)

B. Question about the use of antimicrobial drugs

(This information will be used for consideration of AMR countermeasure promotion committee of the Association)

Question 10. Please provide all antimicrobial drugs (all the veterinary, human, and imported drugs) used in your clinic in a year between 2017 April 1 and 2018 March 31. (Please photocopy the sheet and add if the rows are not enough)

	Category (choose one)	Trade name	Route (choose one)	Weight of component (with unit)	Annual usage (with unit)
1	Veterinary<br>Human<br>Imported		Oral, injection, insertion, eye drop, ointment, others ( )		
2	Veterinary<br>Human<br>Imported		Oral, injection, insertion, eye drop, ointment, others ( )		
3	Veterinary<br>Human<br>Imported		Oral, injection, insertion, eye drop, ointment, others ( )		
45	Veterinary<br>Human<br>Imported		Oral, injection, insertion, eye drop, ointment, others ( )		

Notes for answering:

(1) For category, please put a circle on either veterinary, human, or imported drugs.

(2) For trade name, please describe exact trade name as sold. In case of generic drug indicated as (general name + type (tablet or capsule) + weight + company name), describe as it is.

(3) For route, please put a circle on either oral, injection, insertion, eye drop, ointment, and others. If you choose others, please provide the detail.

(4) For weight of component, please describe the weight per unit, together with the unit, like (aa mg/1 tablet), (bb mg/1 vial), and (cc mg/1 gram).

(5) For annual usage, please describe like (how many boxes which contains how many tablets), so that total annual drug usage can be calculated (together with (4), bulk conversion volume will be calculated).

* Hereafter, questions on microchip follow, but they are out of the purpose of this publication, and will be omitted from this translation.

Thank you for your participation.
Please return the response in the return envelope attached to:
Japan Veterinary Association [Companion animal clinic survey]

Inquiry about the survey:
Japan Veterinary Association [Companion animal clinic survey]
(Attention to Mr. Matsuoka)
Shin-Aoyama building West 23 F, 1-1-1 Minami Aoyama, Minato-Ku, Tokyo, 107-0062
Tel: 03-3475-1601/ Fax: 03-3475-1604
